# Contrast-enhanced ultrasound findings of primary hepatic non-Hodgkin’s lymphoma: a case report

**DOI:** 10.3389/fonc.2024.1380392

**Published:** 2024-07-02

**Authors:** Can Tang, Heqing Zhang, Mei Tian, Yulan Peng

**Affiliations:** ^1^ Department of Medical Ultrasound, West China Hospital, Sichuan University, Chengdu, China; ^2^ Department of Medical Ultrasound, Shangjin Nanfu Hospital, Sichuan University, Chengdu, China

**Keywords:** contrast-enhanced ultrasound (CEUS), primary hepatic lymphoma (PHL), non-Hodgkin’s lymphoma (NHL), Imaging diagnosis, case report

## Abstract

Primary hepatic lymphoma (PHL) is rare, and its early diagnosis is difficult. This article presents a primary hepatic non-Hodgkin’s lymphoma (NHL) case report. A 52-year-old woman was admitted to the hospital due to a fever. After undergoing laboratory examination, contrast-enhanced computed tomography (CT), ultrasound, and contrast-enhanced ultrasound (CEUS), only CEUS suggested malignancy. Then, the patient underwent a laparoscopic liver biopsy, which diagnosed NHL. Previous studies have shown that hepatic lymphoma is a hypoglycemic tumor, and the enhanced CT and magnetic resonance imaging (MRI) scans are mostly mildly intensified. At the same time, the two-dimensional and color Doppler ultrasonography are mostly atypical. CEUS has unique advantages in displaying micro-vessels, which can be helpful in the diagnosis of primary hepatic lymphoma.

## Introduction

PHL is a rare disease that accounts for 0.4% of extratodular non-Hodgkin lymphomas and 0.016% of all non-Hodgkin lymphomas (1). Due to its atypical clinical manifestations and imaging findings, the patient’s condition is often delayed (2). Providing valuable imaging information before the intervention is helpful for the treatment of the disease. There are more CT and MRI reports in PHL but fewer on ultrasound and CEUS. Here, we report a case of primary non-Hodgkin lymphoma with emphasis on ultrasound imaging and CEUS.

## Case presentation

**Figure 1:**
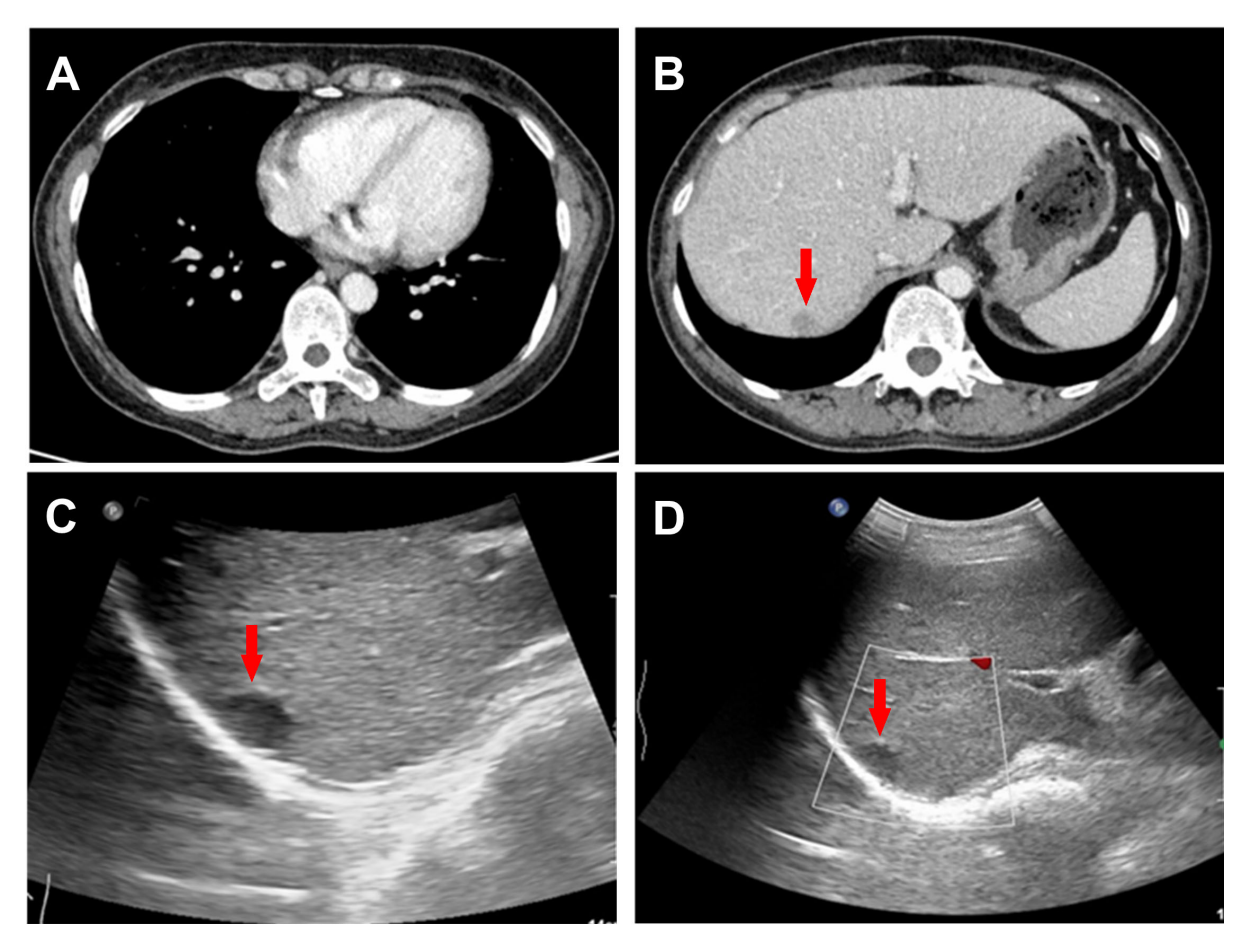
CT presented thickened interlobular septa in both lungs **(A)** and a 1.1cm hypodense nodule in the right upper posterior lobe of the liver **(B)**. Conventional ultrasound revealed a 1.4 × 1.1cm hypoechoic nodule in the upper segment of the right posterior lobe of the liver **(C, D)**.

The patient, a 52-year-old female, was admitted to the hospital due to persistent afternoon fever and fatigue lasting over a month. Her body temperature exhibited fluctuations ranging from 37.1-38.2°during the afternoon and night, accompanied by a cough and a small amount of phlegm. She was admitted to lower-level hospital for treatment. Patient clinical conditions deteriorated, in absence of a clear diagnosis and she was transferred to our hospital. After hospitalization, the whole set before blood transfusion(-), aspartate aminotransferase: 80IU/L (<35IU/L), lactate dehydroase:947IU/L (110-220IU/L), alpha-fetoprotein:7.02ng/ml(<7ng/ml), CA15-3: 27.49U/ml(<24U/ml), serum CA-125: 19.18U/ml (<24U/ml), serum CA19-9: 7.86U/ml(<30U/ml), enolase: 45.38ng/ml(<20.4g/ml), C-reactive protein: 20.80mg/L(<5mg/L), interleukin-6: 47.99pg/ml(0-7pg/ml).Procalcitonin:0.09ng/ml(<0.046ng/ml), pH:7.45, PCO2: 29.5mmHg; PO2: 63mmHg; SO2:93%. Mycoplasma pneumonia antibody: titer 1:40 positive (+), titer 1:80 positive (+), titer 1:160 positive (+), titer 1:320 retained (+/-), Mycoplasma pneumoniae antibody (agglutination) positive (+), CD4 cell subpopulation 50.85%, cryptococcal antigen titer detection: Negative, immunoglobulin GAM(IgG, IgA, IgM), rheumatoid factor (RF), complement C3, C4, B factor: complement C3 0.7690g/L, complement C4 0.1360g/L, VTE score: 1; Sputum and pharyngeal bacteria culture did not show obvious abnormalities. Chest CT showed multiple nodules in both lungs, mainly in the upper lobes of both lungs, which were suspected to be inflammatory nodules. Both lungs had mild inflammation with thickened interlobular septa, which were alleged to be mild pulmonary oedema (**Figure 1A**). A contrast-enhanced CT of the whole abdomen showed multiple mild enhancement nodules and patchy shadows in the renal cortex, which were supposed to be caused by infection. A slightly hypodense nodule with a long diameter of 1.1cm was found in the right upper posterior lobe of the liver, which was mildly enhanced and suspected of inflammation (**Figure 1B**).

Based on the patient’s clinical symptoms, imaging and laboratory results, clinicians first consider infectious lesions. Moxifloxacin was used as an anti-infective therapy after discussion with patients and their families; however, it did not produce satisfactory results. Combined with the follow-up examination results: The results of the antihistone antibody and anti-mitochondrial antibody tests were inconclusive, while the anti-dSDNA and antinuclear antibody assays showed suspicious (±) results for both; thyroid function: Triiodothyronine was 0.91nmol/L, free triiodothyronine was 1.84pmol/L, thyroxine was 40.30nmol/L, and free thyroxine was 5.67pmol/L. ANCA antibody profile (6 items) showed no significant abnormality. Considering the blood-derived infection, the clinician adjusted the antibiotics to Piperacillin/+minocycline/doxycycline for anti-infective treatment, but the patient still had a recurrent fever. Combined with the detection of Staphylococcus aureus, pneumocystis carinii and human herpes virus type 5(HHV-5) in the patient’s alveolar perfusion fluid next-generation sequencing(NGS), the antibiotics were adjusted to meropenem+vancomycin, and the patient did not have fever after treatment with compound sulfamethoxazole against pneumocystis. Consider patients with pneumocystis pneumonia, and give meprednisolone anti-inflammatory therapy. No bacterial growth was found in subsequent blood culture, identification and anaerobic culture. However, the patient still had intermittent low fever and was found to be positive for novel coronavirus COVID-19 during late hospitalization. The attending doctor recommended that the patient undergo a PET-CT examination to identify the cause of the fever further, but the patient refused due to his financial situation and radiation.

Additional ultrasonography indicated no abnormal growth of lymph nodes in the neck, supraclavicular region, axilla, abdominal cavity and retroperitoneum. Routine ultrasound examination showed a 1.4×1.1cm hypoechoic nodule under the upper capsule of the right posterior lobe of the liver, with clear boundaries, regular shape and no blood flow signal (**Figure 1C, D**). The patient’s liver nodules did not disappear under the treatment of anti-inflammatory drugs. Considering that clarifying the nature of the liver may be a diagnostic breakthrough for the patient, contrast-enhanced ultrasound is recommended to describe the nature and evaluate the path of percutaneous liver puncture to obtain a pathological diagnosis, and the patient’s consent is obtained.

**Figure 2:**
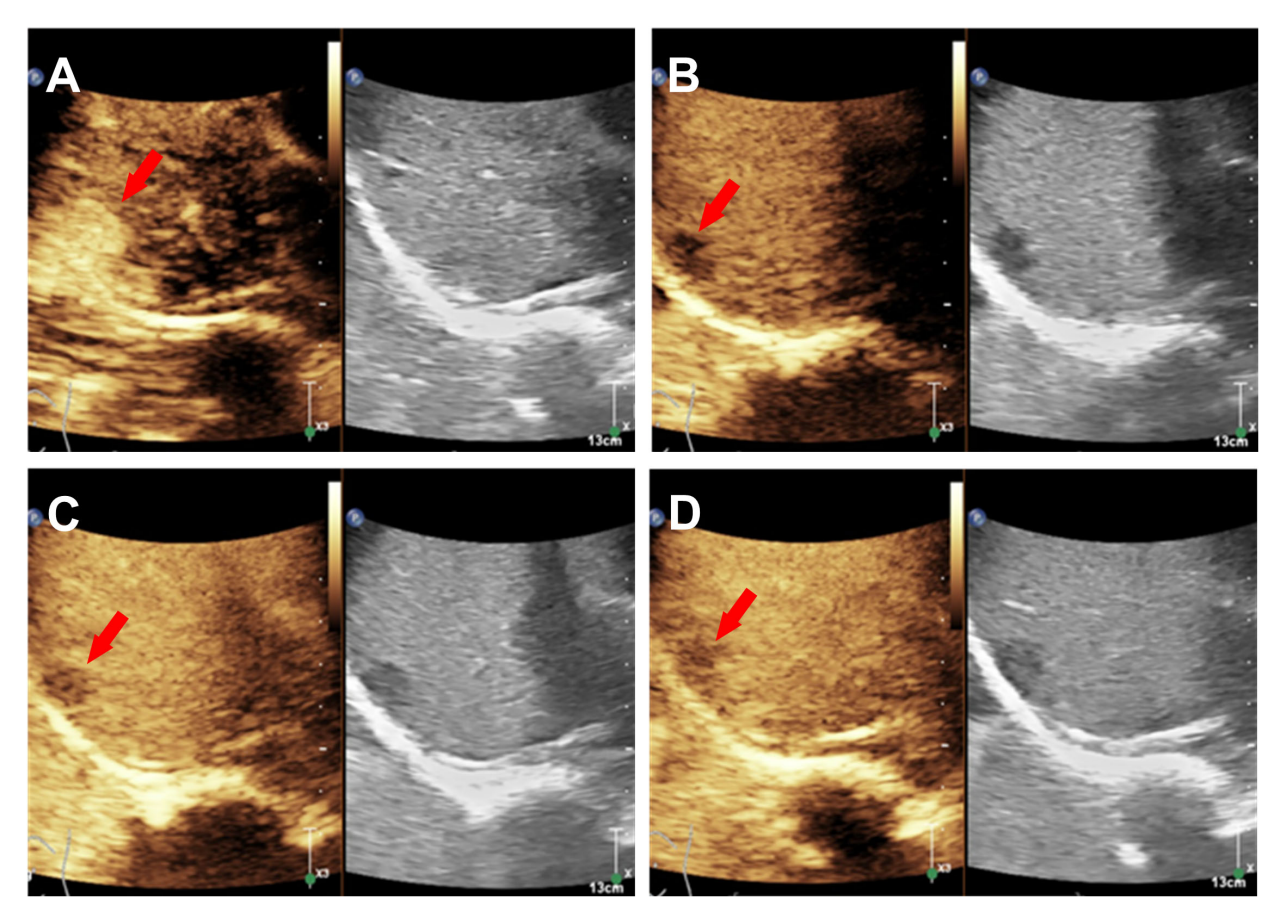
CEUS showed that the uniformly hyper-enhanced solid component of the nodule in the arterial phase **(A)**, the eluted enhancement in the late arterial **(B)** and the slight low enhancement in the portal and parenchymal phases **(C, D)**.

**Figure 3:**
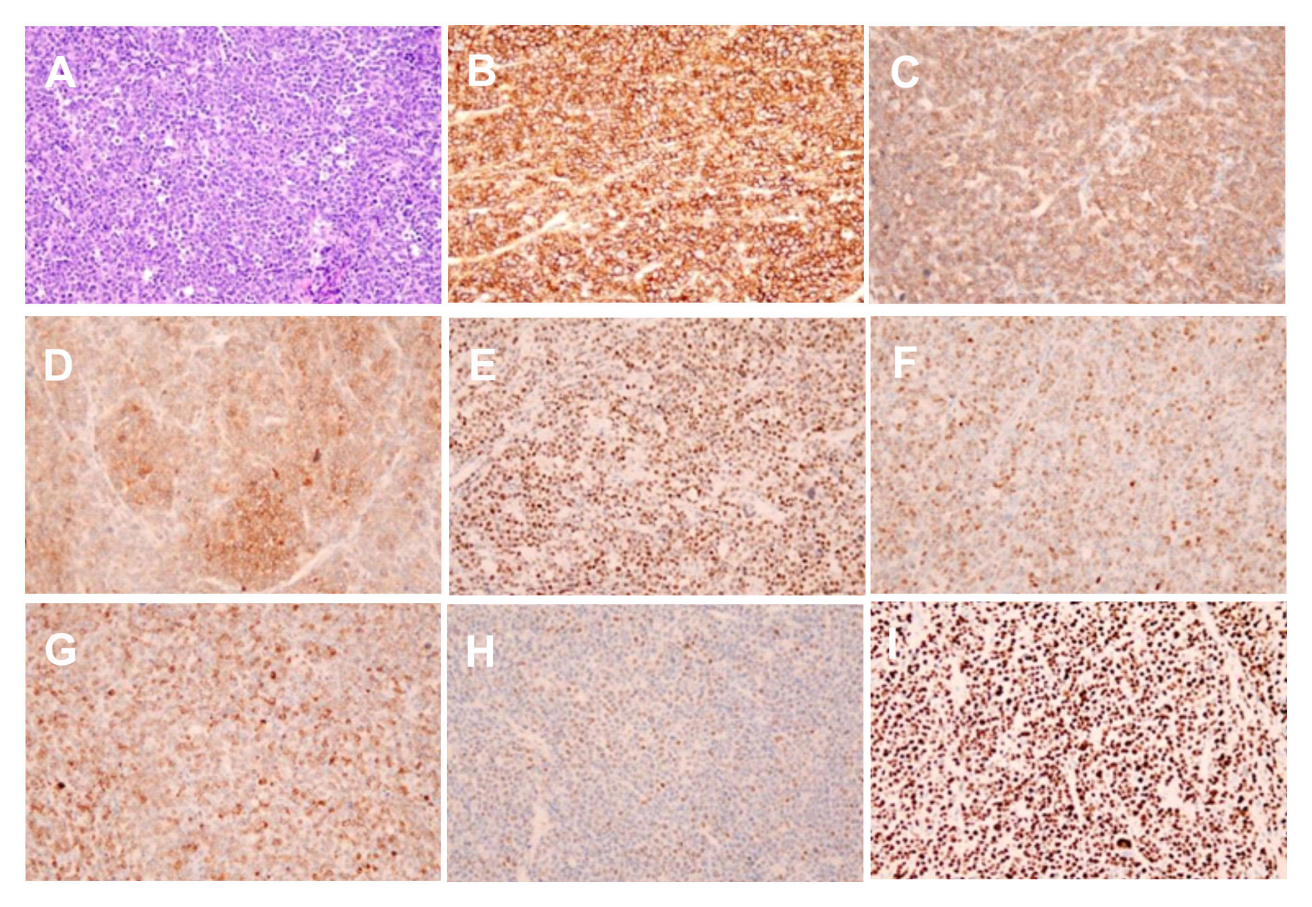
Microscopy showed the HE staining **(A)** (X200) and the immunohistochemical staining of the CD20(+), CD79a(+), CD10(+), Bcl6(+), MUM1(+), Bcl2(+), c-myc(+) and Ki67(+) expression **(B-I)** (X200).

To achieve the best diagnostic display results, the ultrasound machine was adjusted according to the doctor’s habits. A 1.5 ml ultrasound contrast agent SonoVue (Bracco, Milan, Italy) suspension and 5 ml saline were injected successively along the left cubital vein to achieve the purpose of angiography and flushing the tube. The target lesion and liver parenchyma were observed continuously for 5 minutes after the injection. According to the guidelines, the arterial, portal, and delayed phases were defined as 10 to 30 seconds, 30 to 120 seconds, and 121 to 360 seconds after injection, respectively (3). All results were recorded on the ultrasound system. In the arterial phase, the solid component of the nodule rapidly becomes uniformly hyper-enhanced, and the scope becomes larger after enhancement (**Figure 2A**). Then, the enhancement washout begins in the late arterial phase (**Figure 2B**). The portal and parenchymal phases showed slightly less enhancement (**Figure 2C, D**). No unenhanced region was found in the three stages of the lesion. Unlike the inflammation suggested by enhanced CT diagnosis, CEUS is similar to the “fast in and fast out” enhancement model of hepatic malignant tumors. A biopsy was performed to confirm the diagnosis. Ultrasound-guided percutaneous liver biopsy is difficult because of the high location and small size of the tumor. So, the selection of laparoscopic biopsy under general anaesthesia was made to determine the nature and possible origin of the nodules. Intraoperative freezing indicated a potential tumor, leading to the performance of laparoscopic resection on the right hepatic mass. Most postoperative paraffin sections revealed neoplasms originating from the lymphopoietic system. An immunohistochemical study showed that the tumor cells were positive for CD20, CD79a, CD10, Bcl-6, Bcl-2, c-Myc and MUM1 (**Figure 3**). The positive rate of Ki-67(MIB-1) is about 70%. IgH and IgK gene rearrangement tests were positive, and other results were negative. These findings, together with the morphology, support a diagnosis of NHL, and the subtype was diffuse large B-cell lymphoma that favors a non-germinal center B-cell origin. Combined with ultrasonography and CT examination results, no other lesions were suggested, and primary hepatic lymphoma was considered. Subsequently, the patient was referred to the hematology department for chemotherapy with the DA-EPOCH regimen.

## Discussion

Liver lymphoma can be divided into two types: primary and secondary (4). Although the gastrointestinal tract is the most common site of metastasis in NHL patients, half of these patients can metastasize to the liver (5),In contrast, primary hepatic lymphoma is rare, less than 1% (6).

The exact cause of primary hepatic NHL is unclear. It may be related to immunosuppressive agents and hepatitis virus infection and may also occur in healthy people (7, 8). Its most common subtype is diffuse large B-cell lymphoma (9). The patient was diagnosed with fever, had no history of hepatitis virus infection, and was complicated with Staphylococcus aureus pneumonia and pneumocystis pneumonia. The positive nucleic acid of the novel coronavirus was found in the later admission stage, and it could not be determined whether it was a nosocomial infection. The condition was complex, and the clinical manifestations were non-specific. The liver lesion was found by chance, and the cause of the disease may be related to the patient’s autoimmune function (10). Regarding experimental examination, CEA was negative, AFP was at critical value, AST was increased 2-3 times, and LDH was significantly increased, consistent with previous reports (11).

Imaging, though, plays a crucial role in diagnosis. The CT report was consistent with previous reports, showing slightly low-density shadows with clear boundaries and mild enhancement by enhanced scan (12). However, this patient did not have the characteristic features of the “vascular floating sign” and “target ring sign” characteristic for the diagnosis of lymphoma, so it could not be distinguished from the CT findings of inflammation and necrosis (13).

The ultrasonographic manifestations of hepatic NHL are single, multiple or diffuse infiltration, which is more common in Asian patients, and most of the lesions were hypoechoic and lacked blood supply (14). In this case, ultrasound showed a small low-echo mass in the liver capsule with clear boundaries and no abundant blood flow signals. Two-dimensional ultrasound and Doppler ultrasound showed no specificity, while the contrast-enhanced ultrasound demonstrated malignant features consistent with the “fast forward and fast out” pattern, which is in line with previous findings (15). However, it should be distinguished from hepatocellular carcinoma and inflammatory pseudotumor of the liver (16). Hepatocellular carcinoma mainly occurs in the background of liver injury or cirrhosis. Typical small hepatocellular carcinoma is characterized by rapid global enhancement in the arterial phase, low enhancement in the portal and parenchymal phases by contrast-enhanced ultrasound, and inflammatory pseudotumor of the liver can show inflammatory processes, such as increased C-reactive protein (17). The patient, in this case, was originally co-infected. It could not be identified, and CEUS could also be similar to this patient, but the degree of arterial enhancement was slightly higher or equivalent to the peripheral tissue. In this case, the enhancement range of arterial stage lesions increased, and no enhancement was seen after clearance, which has not been reported in the past.

Hepatic lymphoma originates from hepatic interstitial cells, and the increase in arterial phase may be related to the micro-infiltration of the tumor into the surrounding area. However, the clearance was not obvious (18). It may also be because the tumor is small, located under the liver capsule, and the high microbubble concentration has strong backwards-reflecting signals and specular artefacts (19). The specific reasons need to be further verified. Although CEUS could not directly indicate lymphoma for such small liver lesions, when other imaging examinations were of limited help, the malignant signs presented by CEUS showed its unique advantages, providing valuable diagnostic information for the clinic and helping the selection of the biopsy area, contributing to the further diagnosis and treatment of patients (20).

There is no uniform standard for treating primary hepatic non-Hodgkin lymphoma (21). Surgery and chemoradiotherapy are both available options (22). Surgical resection of localized lesions was insufficient, and extrahepatic recurrence might occur. Therefore, the patient was treated with chemotherapy in the hematology department after surgery. At present, he underwent stage 4 chemotherapy with the DA EPOCH (etoposide, prednisone, vincristine, cyclophosphamide and doxorubicin) regimen and survived well (23).

In conclusion, Contrast-enhanced ultrasound combined with experimental examination may provide valuable diagnostic information for isolated small hepatic lymphoma. Primary but hepatic lymphoma is not specific in clinical or radiographic examination, and the diagnosis depends on pathological diagnosis (24).

## Data availability statement

The raw data supporting the conclusions of this article will be made available by the authors, without undue reservation.

## Ethics statement

The studies involving humans were approved by Bioethics Committee of West China Hospital of Sichuan University. The studies were conducted in accordance with the local legislation and institutional requirements. The participants provided their written informed consent to participate in this study. Written informed consent was obtained from the individual(s) for the publication of any potentially identifiable images or data included in this article.

## Author contributions

CT: Methodology, Writing – original draft, Writing – review & editing. HZ: Supervision, Validation, Writing – review & editing. MT: Data curation, Formal analysis, Resources, Writing – original draft. YP: Resources, Supervision, Validation, Visualization, Writing – review & editing.
